# A six-week parenting program for parents of children with learning disabilities in Greece

**DOI:** 10.1093/eurpub/ckac131.499

**Published:** 2022-10-25

**Authors:** N Pedioti, M Papadakaki, K Koutra, S Parlalis

**Affiliations:** 1 Department of Social Work, LaHeRS & PHC-HMU, Hellenic Mediterranean University, Heraklion, Greece; 2 Department of Psychology, University of Crete, Heraklion, Greece; 3 Department of Social Work, Frederick University, Limassol, Cyprus

## Abstract

Children with learning disabilities have been shown to experience complex emotional and conduct difficulties due to late diagnosis and inappropriate parental management. Parenting support have been deemed necessary for a holistic management of learning disabilities. The current presentation reports on the methodology and initial results of a program offered to parents of children aged 8-12 years with learning difficulties at the region of Crete in Greece, during the school year 2020-2021. The program aimed at improving parenting skills and parent-child interaction, through a short training (four meetings) and a set of seven techniques offered to parents for boosting children’s positive response. All children diagnosed with a learning disability by an interdisciplinary team of experts serving a mobile unit, were approached and their parents were offered a personalised intervention plan and a package of counselling services including a six-week program, based on the “A Six Week Parenting Program For Child Compliance” (Cipani, 2005). Parents were randomly assigned to an intervention (n = 40, 6-week parenting program) and a control group (n = 29, no intervention). A pre- and post evaluation design, with self-administered questionnaires, was used to assess changes in parental skills and performance as well as the quality of parent-child interaction (Parenting Style Questionnaire; the Family Quality of Life Scale (FQOL)). Most of the participants were mothers (n = 63), of low educational level (n = 26) and low income (n = 49). Statistically significant improvements were evident in the intervention group at post-intervention level in parenting skills as compared to baseline scores. Significant differences were also observed in the parenting skills at post-intervention level, between the intervention and the control group. Findings are expected to highlight new approaches towards improving the parental role, with an impact on the quality of life of families.

**Key messages:**

• Training on parenting skills should be included in family support services offered to children with learning disabilities.

• Professionals working with learning disabilities should be trained to assess deficits in parenting skills and offer brief counseling to parents.

